# Dihydrosanguinarine: A Review of Its Pharmacology, Structure–Activity Relationship, Toxicity, Pharmacokinetics, and Clinical Prospects

**DOI:** 10.3390/ijms27114852

**Published:** 2026-05-28

**Authors:** Xiaoqi Yin, Yingyi Cao, Chuxuan Fang, Ce Zhang, Jiaming Yang, Mingyu Yu, Gong Cheng, Long Yang

**Affiliations:** 1 School of Integrative Medicine, Tianjin University of Traditional Chinese Medicine, Tianjin 301617, China; 13555643164@163.com (X.Y.);; 2 New Cornerstone Science Laboratory, Tsinghua University-Peking University Joint Center for Life Sciences, School of Basic Medical Sciences, Tsinghua University, Beijing 100084, China; yy-cao22@mails.tsinghua.edu.cn; 3 School of Medical Technology, Tianjin University of Traditional Chinese Medicine, Tianjin 301617, China; 15981777628@163.com (C.F.); yumy@tjutcm.edu.cn (M.Y.); 4 Department of Anatomy, School of Basic Medicine Sciences, Tianjin Medical University, Tianjin 300070, China; 5 Research Center for Infectious Diseases, Tianjin University of Traditional Chinese Medicine, Tianjin 301617, China; 6 School of Public Health, Tianjin University of Traditional Chinese Medicine, Tianjin 301617, China

**Keywords:** dihydrosanguinarine, pharmacological activities, molecular mechanism, toxicity, pharmacokinetics, structure–activity relationship

## Abstract

Dihydrosanguinarine (DHSA) is a naturally occurring benzo[c]phenanthridine alkaloid primarily isolated from plants of the Papaveraceae family. DHSA exhibits broad pharmacological activities, including antitumor, anti-inflammatory, hypoglycemic, neuroprotective, analgesic, anxiolytic, antiarrhythmic, and antimicrobial effects. Mechanistically, DHSA regulates multiple signaling pathways and molecular targets, including TMEM16A, p53, Ras/Raf/MEK/ERK, PI3K/AKT, NF-κB, PPARγ, GABA_A_ receptors, and voltage-gated sodium channels. Compared with its biosynthetic precursor sanguinarine (SA), DHSA exhibits a comparatively favorable safety profile while retaining considerable biological activity. Pharmacokinetic studies further suggest that DHSA possesses acceptable membrane permeability, gastrointestinal absorption potential, enterohepatic circulation characteristics, and sustained systemic exposure. In addition, structure–activity relationship (SAR) and electrostatic surface potential (ESP) analyses indicate that the chemically accessible C6 position may provide opportunities for rational structural optimization. Nevertheless, the clinical translation of DHSA still faces several challenges. Therefore, this review systematically summarizes the physicochemical properties, pharmacological activities, molecular mechanisms, SAR characteristics, ESP distribution, toxicity, pharmacokinetic behavior, and clinical prospects of DHSA, aiming to provide a theoretical basis for its future drug development and translational application.

## 1. Introduction

Since the 1950s, natural products derived from medicinal plants have served as a valuable source of innovative therapeutic agents for human use [1]. With the advancement of science and technology, an increasing number of plant extracts have been identified as possessing practical medical value [2]. Moreover, the sources for these compounds are readily available, and the corresponding manufacturing processes are characterized by simplicity and sustainability. Numerous scientific studies have focused on the extraction of active monomers from medicinal plants, many of which—such as paclitaxel and artemisinin—have been widely applied in clinical practice [3,4]. According to statistical data, over 60% of currently marketed drugs are directly or indirectly derived from natural products. These natural products can also serve as lead compounds for structural optimization and derivatization to enhance their medicinal potential [5]. Thus, increased attention to natural product research is warranted.

**Figure 1:**
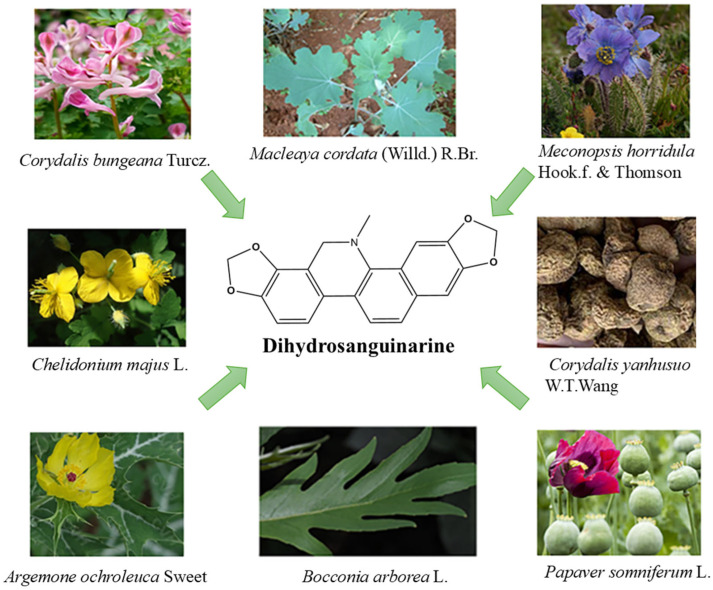
Natural plant sources of dihydrosanguinarine (DHSA). Green arrows indicate botanical origins from which dihydrosanguinarine has been isolated or identified.

Dihydrosanguinarine (DHSA), with the chemical formula C_20_H_15_NO_4_, is a natural product featuring a phenanthridine ring structure incorporating an isoquinoline nucleus as its central framework. As a typical isoquinoline alkaloid, DHSA was initially extracted from Argemone oil by S. N. Sarkar in 1948 and can be effectively isolated using organic solvents such as ethanol and ethyl acetate [6,7]. It is primarily derived from the aerial components of Papaveraceae species and can also be synthesized via biological and chemical methods [8,9]. DHSA appears as white to pale yellow crystalline solids and is predominantly sourced from *Corydalis bungeana* Turcz., *Chelidonium majus* L. (*C. majus*), *Macleaya cordata* (Willd.) R.Br., *Bocconia arborea* L., *Argemone ochroleuca* Sweet, and *Papaver somniferum* L. Supplementary botanical sources comprise *Corydalis yanhusuo* W. T. Wang and *Meconopsis horridula* Hook. f. & Thomson [7,10,11,12,13,14] (see Figure 1). The *C. majus* extracts were used in European folk medicine for anti-inflammatory and analgesic effects, while *Macleaya cordata* has been integrated into veterinary medicine for its antibacterial properties [10].

In recent years, an increasing number of studies on DHSA have been published, revealing its diverse pharmacological activities, including antitumor, anti-inflammatory, hypoglycemic, neuroprotective, and antimicrobial effects [7,10,15,16,17]. Preliminary pharmacokinetic studies have suggested that DHSA is the primary metabolite of sanguinarine (SA) formed in vivo [18]. Compared with its precursor compound SA, DHSA exhibits a more favorable safety profile while retaining considerable biological activity [19]. However, to date, a comprehensive review of this promising natural product remains lacking. Therefore, this review systematically summarizes and integrates the current evidence regarding the physicochemical properties, pharmacological activities, molecular mechanisms, structure–activity relationship (SAR) characteristics, electrostatic surface potential (ESP) distribution, toxicity, and pharmacokinetic behavior of DHSA. In addition, the major challenges associated with its clinical translation, including multitarget-related safety concerns, pharmacokinetic limitations, drug–drug interaction risks, and pharmaceutical development bottlenecks, are further discussed. Finally, future perspectives involving rational structural modification and precision medicine strategies are proposed in order to provide a theoretical basis for the future development of DHSA as a clinically valuable therapeutic candidate or lead compound.

## 2. Physicochemical Properties of DHSA

DHSA, with the chemical formula C_20_H_15_NO_4_, is a benzophenanthridine alkaloid possessing a tetracyclic benzo[c]phenanthridine core. Its structure is characterized by a neutral, tetrahydro-nitrogen atom within the partially saturated C ring, whereas rings A, B, and D are fully aromatic. Two methylenedioxy bridges are fused to the aromatic system at the C2–C3 and C8–C9 positions, respectively. The molecular identity of dihydrosanguinarine is defined by this specific arrangement of fused rings, the reduced nitrogen center (C6), and the two methylenedioxy substituents. DHSA is soluble in organic solvents such as methanol, ethanol, and DMSO, and appears as white to pale yellow crystalline solids. It has a molecular weight of 333.3 g/mol and a density of 1.426 g/cm^3^. Additional physicochemical properties of DHSA are summarized in Table 1.

## 3. Pharmacological Effects of DHSA

**Figure 2:**
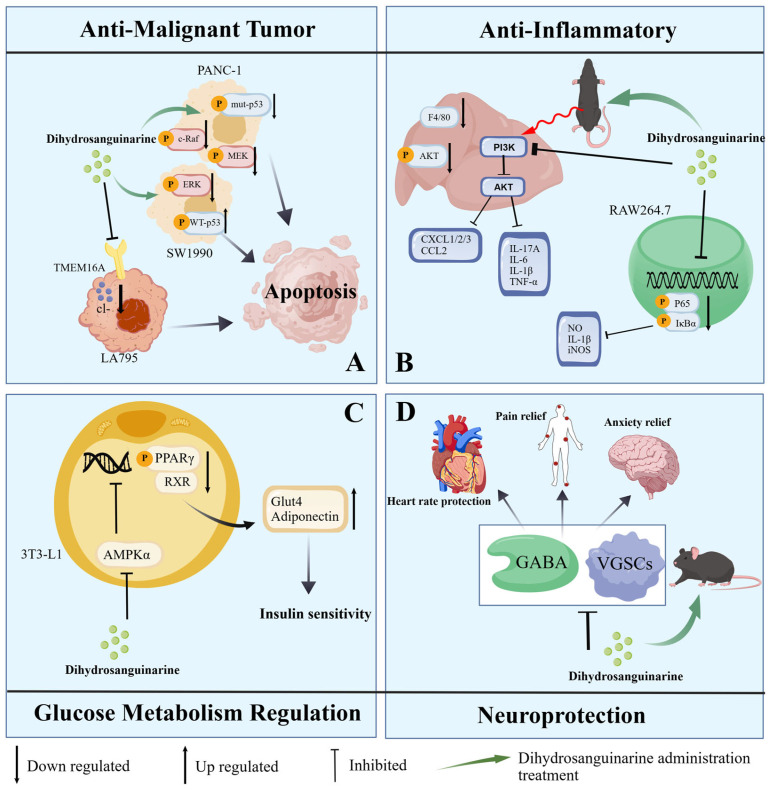
(**A**) Dihydrosanguinarine (DHSA) primarily exerts its anti-tumor effects by inducing apoptosis in LA795, SW1990 and PANC-1 cells. (**B**) DHSA inhibits the PI3K/AKT signaling pathway to suppress LPS-induced hepatitis in mice. In addition, DHSA also inhibits the NF-κB pathway and exerts anti-inflammatory effects in vitro. (**C**) DHSA influences the AMPK signaling pathway to enhance the body’s insulin sensitivity. (**D**) DHSA primarily influences the neuroprotective effects of VGSCs and GABA receptors. Created with BioGDP.com (https://biogdp.com) (accessed on 14 February 2026).

DHSA demonstrates excellent anti-malignant tumor, anti-inflammatory, glycemic regulation, neuroprotection and antibacterial effects, making it a promising small-molecule candidate worthy of further development. This section synthesizes DHSA’s pharmacological activities and mechanisms (Table 2). The primary pharmacological mechanism of DHSA is shown in Figure 2.

### 3.1. Anti-Malignant Tumor Effect

DHSA primarily antagonizes the endogenous TMEM16A current in cells, modulates intracellular p53 in a bidirectional manner, and suppresses the rat sarcoma (Ras)/raf kinase (Raf)/mitogen-activated protein kinase kinase (MEK)/extracellular regulated protein kinase (ERK) signaling cascade. These actions collectively inhibit cancer cell proliferation and migration, and promote apoptosis. The anti-malignant tumor mechanism of DHSA is illustrated in Figure 2A.

#### 3.1.1. Anti-Lung Adenocarcinoma

Lung adenocarcinoma (LUAD), the predominant subtype of lung cancer, accounts for approximately 35–40% of all lung cancer cases [23]. TMEM16A is overexpressed in several malignancies and plays a crucial role in carcinogenesis [24,25]. Experimental evidence demonstrates that DHSA exerts a dose-dependent inhibition of TMEM16A channel activity and attenuates endogenous TMEM16A currents, thereby efficiently suppressing the growth of lung adenocarcinoma cells (LA795). Moreover, within the concentration range of 1–10 μM, DHSA significantly impairs LA795 cell motility and promotes apoptosis [20].

#### 3.1.2. Anti-Pancreatic Cancer

Pancreatic cancer, characterized by exceptionally high incidence and mortality rates among cancers, remains notably limited in treatment alternatives [26,27]. The p53 protein is crucial for cell cycle regulation and apoptosis; it induces cell cycle arrest at the G1-S or G2-M phases by downregulating cell division cycle 25C (Cdc25c), a phosphatase that hinders cell cycle progression, and by upregulating p21, a cyclin-dependent kinase (CDK) inhibitor [28,29]. DHSA demonstrates selective anti-proliferative effects on pancreatic cancer cell lines (PANC-1 and SW1990) via mechanisms distinct for each cell line. In PANC-1 cells, it diminishes the overexpression of mutant p53 (mut-p53) to reinstate normal regulation of G0/G1 and G2/M phase transitions while simultaneously activating the mitogen-activated protein kinase (MAPK) signaling pathway to trigger apoptosis. In SW1990 cells, DHSA upregulates the expression of wild-type p53 (WT-p53) to augment its tumor-suppressive function [15]. Moreover, DHSA has been shown to inhibit Ras and its downstream effectors, including p-Raf, p-MEK, and p-ERK, thereby effectively obstructing the activity of this signaling cascade. This molecular intervention results in reduced pancreatic cancer cell proliferation, suppressed invasive capability, and increased apoptosis [15].

### 3.2. Anti-Inflammatory Effect

In an LPS-induced hepatitis male Balb/c mice model, DHSA modulates hepatic macrophage activity by attenuating their aberrant activation and infiltration, leading to the synergistic suppression of three key pro-inflammatory signaling cascades—namely the TNF, IL-17, and phosphatidylinositol 3-kinase (PI3K)/protein kinase B (AKT) pathways [10]. This results in decreased expression of downstream pro-inflammatory mediators, including cytokines such as TNF-α, IL-1β, IL-6, and IL-17A, as well as chemokines including C-X-C motif chemokine ligand 1(CXCL1), CXCL2, CXCL3, and CCL2 [10]. In addition, DHSA inhibits the expression of adhesion molecules, thereby collectively contributing to the alleviation of LPS-induced inflammatory injury in the liver [10,30]. Notably, the PI3K/AKT pathway emerges as a critical signaling node in this regulatory process [10,31,32]. The anti-hepatitis mechanism of DHSA in mice is shown in Figure 2B.

*Corydolis bungeana* Turcz. serves as a principal botanical source of DHSA. In vitro, using LPS-stimulated RAW 264.7 murine macrophages, treatment with *Corydalis bungeana* Turcz. Extract significantly suppressed the secretion of pro-inflammatory cytokines including tumor necrosis factor-α (TNF-α), IL-1β, and IL-6 and inhibited the phosphorylation of key nuclear factor kappa-B (NF-κB) pathway proteins, namely IκBα and p65 [21]. In vivo, in an LPS-induced BALB/c mouse model, the extract similarly reduced the serum levels of NO and IL-1β, and downregulated both mRNA and protein expression of iNOS and IL-1β in liver tissue [21]. These findings suggest that DHSA may mediate potent anti-inflammatory effects through inhibition of inhibitor of nuclear factor κB alpha (IκBα) and p65 phosphorylation within the NF-κB signaling cascade, leading to the downstream suppression of pro-inflammatory mediators including NO, TNF-α, IL-1β, and IL-6 [33,34].

Collectively, DHSA primarily mediates its anti-inflammatory activity through attenuating inflammatory cell infiltration and modulating key signaling pathways, including the TNF-IL-17-PI3K/AKT axis and NF-κB pathway via inhibition of IκBα and p65 phosphorylation, thus downregulating the production of pro-inflammatory mediators including TNF-α, NO, IL-1β, IL-6, CXCL1/2/3 and CCL2.

### 3.3. Anti-Type 2 Diabetes Effect

Peroxisome proliferator-activated receptor gamma (PPARγ), a member of the nuclear receptor superfamily, has emerged as a pivotal therapeutic target for type 2 diabetes, as its ligands can ameliorate glucose and lipid metabolic abnormalities by increasing insulin sensitivity [35]. Research indicates that DHSA demonstrates dose-dependent antidiabetic effects in vitro. In differentiation trials with 3T3-L1 mouse adipocytes, 5 μM DHSA markedly decreased AMP-activated protein kinase (AMPK) levels, thereby blocking the downstream retinoid X receptor (RXR) pathway and reducing p-PPARγ [16]. This inhibition activates PPARγ receptors, consequently enhancing the production of transcription factors such as adipocyte protein 2 (aP2), cluster of differentiation 36 (CD36), and CCAAT enhancer binding protein alpha (C/EBPα), which facilitates adipocyte differentiation [16,36,37,38]. Simultaneously, PPARγ activation increases the synthesis of insulin-dependent glucose transporter 4 (Glut4) and adiponectin, consequently enhancing glucose uptake efficiency in skeletal muscle and adipose tissue, which in turn amplifies insulin sensitivity [16,39]. The anti-type 2 diabetes mechanism of DHSA is shown in Figure 2C.

In conclusion, by modulating the AMPKα-PPARγ-Glut4 signaling pathway, DHSA effectively lowers blood glucose levels without causing the negative side effects often linked to conventional PPARγ agonists [40]. These findings collectively indicate DHSA’s great potential as a new hypoglycemic agent.

### 3.4. Neuroprotection

DHSA primarily mediates its analgesic, anxiolytic, anticonvulsant, and antiarrhythmic effects through modulation of GABA_A_ receptors, opioid receptors, and alpha-2A adrenergic receptors, as well as intracellular voltage-gated sodium channels Nav1.5 and Nav1.7. The neurological and ion-channel-related mechanisms of DHSA are shown in Figure 2D.

#### 3.4.1. Analgesic Effect

DHSA has been documented to demonstrate considerable analgesic benefits in both neurogenic and inflammatory stages of pain. The concurrent administration of DHSA with the opioid receptor antagonist naltrexone (NTX) or the non-competitive GABA_A_ receptor antagonist picrotoxin (PTX) through intraperitoneal injection in mice led to a reduction in its analgesic efficacy, indicating that DHSA’s mechanism of action may involve the modulation of GABA_A_ and opioid receptors [12]. GABA_A_ receptors are identified as potential therapeutic targets for pain treatment, with clinical trials showing the effectiveness of GABA receptor agonists and inhibitors of GABA uptake or metabolism in mitigating pain symptoms [41,42,43]. Traditional opioid analgesics are linked to gastrointestinal and neurological effects [44,45]. In safety evaluations, DHSA given at analgesic levels did not cause notable drowsiness or other neurotoxic effects and significantly reduced stomach tissue damage generated by absolute ethanol [12].

Voltage-gated sodium channels (VGSCs), comprising nine α-subunits (Nav1.1 to Nav1.9), are prominent therapeutic targets for neurological disorders [46]. Nav1.7 has emerged as a viable therapeutic target in the development of analgesic drugs, with several Nav1.7-targeted inhibitors currently undergoing clinical trials [47]. Molecular docking analyses demonstrated a significant binding affinity between DHSA and Nav1.7, indicated by a binding energy of ΔG = −7.38 kcal/mol [22]. Moreover, whole-cell patch-clamp investigations revealed that DHSA suppressed Na^+^ current peaks driven by Nav1.7 in a concentration-dependent manner, caused a leftward shift in the channel activation curve, and expedited the activation phase [22]. The findings collectively indicate that DHSA exerts its analgesic effects through two mechanisms: the inhibition of Na^+^ currents and the modification of Nav1.7 channel activation kinetics via direct contact with the channel protein.

In summary, DHSA exerts its analgesic effects via a multi-target mechanism that involves the synergistic activation of opioid receptors and GABA_A_ receptors while simultaneously regulating VGSCs. The pharmacodynamic profile not only illustrates the therapeutic rationale of DHSA in pain treatment but also highlights its enhanced safety relative to other analgesics, positioning it as a highly promising therapeutic candidate.

#### 3.4.2. Anxiolytic Effect

The GABA_A_ receptor, a pentameric ligand-gated chloride channel essential for the vertebrate nervous system, primarily regulates inhibitory neurotransmission and functions as a therapeutic target for anesthetics [48]. In the hole-board test, oral administration of DHSA in mice markedly elevated head-dip counts (ED50 = 2 mg/kg), a result analogous to the positive control clonazepam (ED50 = 1.5 mg/kg) [7]. The anxiolytic effect was nullified by prior administration of the GABA_A_ receptor antagonist bicuculline [7]. Molecular docking demonstrated that DHSA interacts with the GABA_A_ receptor through π-π and π-alkyl interactions with critical residues and establishes hydrogen bonds with Ser90 of the α2A-adrenergic receptor, supported by hydrophobic contacts [7,49]. The data indicate that DHSA’s anxiolytic mechanism entails dual regulation of the GABAergic and noradrenergic systems.

#### 3.4.3. Anti-Arrhythmic Effect

Heart failure frequently precipitates arrhythmias, resulting in left ventricular systolic dysfunction (LVSD) and eventual arrhythmia-induced cardiomyopathy (AIC) [50]. Nav1.5, the primary voltage-gated sodium channel subtype in the heart, is associated with several cardiac disorders, including arrhythmias, when mutated [51]. Molecular docking studies indicate that DHSA possesses a robust binding affinity for Nav1.5, with its binding site situated within the channel’s pore-forming region. This interaction directly regulates sodium channel gating, hence affecting the generation and propagation of cardiomyocyte action potentials [22]. In whole-cell patch-clamp tests, DHSA at 10 μmol/L reduced Nav1.5 peak currents by 74% and modified the activation and inactivation kinetics of the channel, effectively regulating its opening and closing dynamics [22]. These regulatory effects may reinstate normal electrical characteristics in cardiomyocytes, hence diminishing arrhythmogenic vulnerability.

### 3.5. Antibacterial Effect

Bacterial and fungal infections have consistently posed a significant hazard to human health, with clinical mortality rates remaining elevated over an extended period [52,53,54]. In this regard, naturally generated isoquinoline alkaloids have garnered significant interest owing to their extensive antibacterial efficacy [55,56]. DHSA exhibits a pronounced inhibitory impact on Staphylococcus aureus and a dose-dependent inhibition of several clinically relevant Candida species, including Candida albicans, Candida parapsilosis, and Candida neoformans [57,58]. Currently, research indicates that the antibacterial mechanism of DHSA primarily operates through two pathways: one involves disrupting microbial DNA replication by inhibiting topoisomerase activity and inducing DNA damage; the other entails compromising cell membrane structural integrity, leading to intracellular ion imbalance and content leakage. Nonetheless, the actual molecular mechanism behind the aforementioned mode of action remains inadequately understood, and further investigation into the structure–activity relationship and the associated signaling pathways is warranted [59].

### 3.6. Comprehensive Pharmacological Insights of DHSA

**Figure 3:**
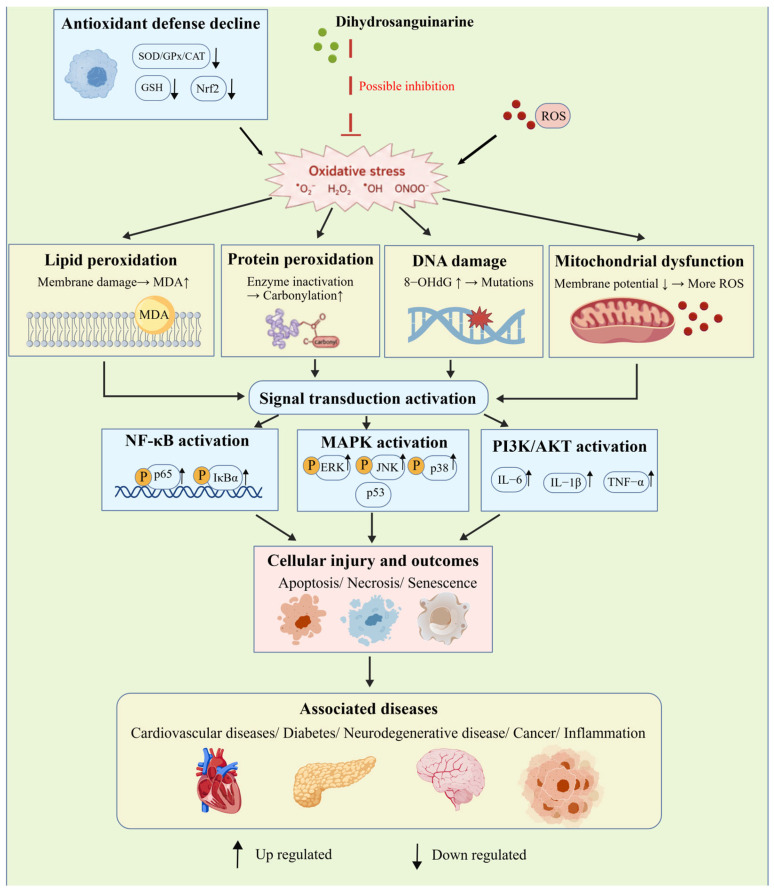
Mechanism of oxidative stress–mediated cellular injury and the potential protective role of dihydrosanguinarine. Oxidative stress is initiated by excessive accumulation of reactive oxygen species (ROS) together with impaired antioxidant defense systems, characterized by decreased activities of antioxidant enzymes (SOD, GPx, and CAT), reduced glutathione (GSH) levels, and suppression of Nrf2 signaling. Elevated oxidative stress subsequently induces lipid peroxidation, protein oxidation, DNA damage, and mitochondrial dysfunction, leading to increased MDA production, protein carbonylation, 8−OHdG formation, mitochondrial membrane potential collapse, and further ROS generation. These pathological events activate multiple downstream signaling pathways, including NF-κB, MAPK, and PI3K/AKT pathways, thereby promoting inflammatory responses, apoptosis, necrosis, and cellular senescence. Ultimately, persistent oxidative injury contributes to the development of cardiovascular diseases, diabetes, neurodegenerative disorders, cancer, and chronic inflammation. Dihydrosanguinarine may exert protective effects by suppressing ROS accumulation and attenuating oxidative stress–related signaling cascades. Created with BioGDP.com (https://biogdp.com) (accessed on 24 May 2026).

Although DHSA has been reported to regulate multiple signaling pathways across different disease models, currently, studies generally describe these effects as separate downstream events, including modulation of p53, NF-κB, inflammatory cytokines, ion channels, and survival-related signaling pathways [15,21]. However, increasing evidence suggests that these signaling cascades are not functionally isolated, but rather constitute interconnected cellular stress-response networks [60,61]. In particular, p53 and NF-κB are recognized as central regulators coordinating apoptosis, inflammatory cytokine production, oxidative stress responses, metabolic adaptation, and cell-survival signaling [62,63]. Therefore, the regulation of p53, NF-κB, PI3K/AKT, Ras/Raf/MEK/ERK, and inflammatory mediators by DHSA partially reflects DHSA’s regulation of the cellular stress response system, rather than independent linear signaling events [64]. Based on the current evidence, we speculate that DHSA does not function solely as a classical single-target ligand directly acting on molecular targets. Instead, DHSA may exert pleiotropic pharmacological effects through coordinated regulation of upstream cellular stress states, including oxidative stress, inflammatory signaling, mitochondrial homeostasis, and cell-survival responses (Figure 3). Such upstream regulatory effects subsequently influence multiple downstream signaling pathways, including p53, NF-κB, PI3K/AKT, and MAPK-related cascades. This hypothesis explains why DHSA exhibits broad pharmacological activities across tumor, inflammatory, metabolic, and neurological disease models.

Nevertheless, the pharmacological mechanisms of DHSA still demonstrate disease-specific characteristics. In tumor models, DHSA primarily regulates pathways associated with apoptosis, proliferation, migration, and cell-cycle progression [15,20]. In inflammatory and metabolic disease models, its effects are more closely associated with cytokine regulation and suppression of inflammatory signaling cascades [10]. In neurological and analgesic studies, ion-channel regulation and neuronal excitability appear to play central roles in mediating the pharmacological effects of DHSA [7]. Interestingly, these apparently distinct mechanisms ultimately converge on common cellular stress-response and survival-regulatory networks, further supporting the multitarget pharmacological properties of DHSA. Therefore, DHSA may therefore represent a multitarget alkaloid capable of simultaneously modulating interconnected pathological pathways.

This multitarget pharmacological profile provides potential therapeutic advantages in complex diseases involving intertwined pathological mechanisms, such as diabetes accompanied by chronic inflammation, cancer-associated inflammatory responses, or neuropathic pain with neuroinflammatory components [65,66,67]. Under such conditions, DHSA potentially functions either as a multitarget single-agent therapy or as a component of combination therapy aimed at simultaneously regulating inflammation, cellular stress responses, metabolic dysregulation, and aberrant survival signaling networks. However, the same pleiotropic regulatory activity may also increase the risk of off-target effects [68]. Furthermore, whether DHSA directly binds multiple independent molecular targets or indirectly regulates downstream signaling cascades through upstream hub mechanisms remains unclear. Therefore, future studies should focus on identifying the primary molecular targets, upstream regulatory hubs, tissue specificity, and biomarker-guided therapeutic windows of DHSA through integrated pharmacological, omics and molecular docking.

## 4. Structure–Activity Relationship (SAR) Analysis of DHSA

**Figure 4:**
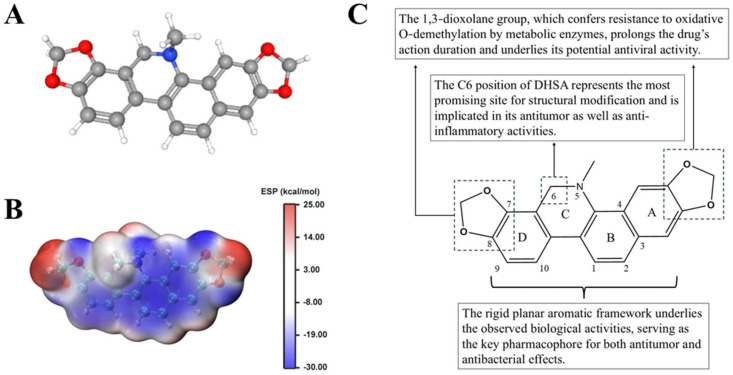
Structural characteristics, electrostatic surface potential (ESP), and structureactivity relationship (SAR) analysis of dihydrosanguinarine (DHSA). (**A**) Three−dimensional molecular structure of DHSA. (**B**) Electrostatic surface potential (ESP) distribution of DHSA. Red regions indicate electron-rich areas with relatively negative electrostatic potential, whereas blue regions represent electron-deficient regions. (**C**) Summary of the major pharmacophoric features and modifiable positions associated with the biological activities of DHSA.

Structurally, DHSA possesses a rigid planar benzo[c]phenanthridine aromatic framework together with two terminal 1,3-dioxolane moieties, both of which are closely associated with its diverse pharmacological activities [7,15] (see Figure 4A). The planar aromatic scaffold enables reversible intercalation between DNA base pairs, thereby interfering with DNA replication, transcription, and repair processes [69]. This mechanism is considered central to the antitumor and antibacterial activities of DHSA. Notably, several established chemotherapeutic agents, including doxorubicin and actinomycin D, as well as certain antibacterial compounds such as pyridoacridines, share similar planar aromatic pharmacophores [70,71].

As summarized in Figure 4C, the rigid planar aromatic framework underlies the broad-spectrum biological activities of DHSA, particularly its antitumor and antibacterial effects [15,17]. In contrast, the terminal 1,3-dioxolane groups contribute not only to biological activity but also to metabolic stability. Previous studies have suggested that methylenedioxy-containing structures confer resistance to oxidative O-demethylation mediated by metabolic enzymes, thereby prolonging drug action duration and enhancing antiviral or antimicrobial activity [72,73]. Moreover, molecules containing 1,3-benzodioxolane structures are widely distributed among natural products and synthetic derivatives with antitumor, antiviral, and antifungal properties [74,75]. These distinct spatial and electronic characteristics may also facilitate the specific interactions of DHSA with some biological targets, thereby influencing the overall pharmacological potency of DHSA.

Interestingly, previous derivatization studies have consistently identified the C6 position as one of the most feasible and pharmacologically responsive regions for structural modification [76,77,78,79]. Unlike the rigid aromatic core directly involved in π–π stacking and DNA intercalation, the C6 position accommodates substituent introduction without severely disrupting the intrinsic planar pharmacophore of DHSA [80]. Therefore, modification at this position may preserve the original biological activity of DHSA while simultaneously introducing new steric, electronic, or lipophilic properties [81]. Studies have shown that introducing a methoxy group at C6, such as in 6-methoxydihydrosanguinarine (6-MDS), enhances antitumor activity while maintaining relatively low neurotoxicity [76,77,78,79,81,82]. Similarly, incorporation of an acetonyl group at C6 (6-acetonyl-5,6-dihydrosanguinarine) activates immune cells through the ROS–ERK/JNK–NF-κB signaling pathway and exerts immunostimulatory effects [83]. Furthermore, alkyl substitution at the C6 position generally increases lipophilicity, thereby favoring transmembrane transport [81].

To further investigate the feasibility of C6 derivatization from an electronic perspective, electrostatic surface potential (ESP) analysis was performed (Figure 4B). The results demonstrated that the terminal methylenedioxy-containing regions exhibited relatively electron-rich negative electrostatic potential distributions, whereas the central aromatic scaffold displayed a comparatively electron-deficient and hydrophobic surface. This polarized electronic distribution facilitates interactions with biological targets through hydrogen bonding, π–π stacking, and hydrophobic interactions, thereby contributing to the broad-spectrum biological activities of DHSA [10]. Interestingly, the electrostatic potential surrounding the C6 position exhibited a moderately positive electrostatic region (~3 kcal/mol), suggesting that this region possesses a chemically accessible and electronically permissive environment for structural derivatization. Such an electronic environment may allow introduction of substituents at the C6 position without severely disrupting the overall electronic distribution or the core planar aromatic pharmacophore responsible for DNA intercalation. Together with previously reported derivatization studies, these findings provide theoretical support for the feasibility of C6-directed structural optimization of DHSA.

Currently, SAR studies of DHSA remain relatively limited, and systematic derivative design combined with high-throughput screening will be important for identifying compounds with improved selectivity and safety profiles [84]. Collectively, the rigid aromatic framework, methylenedioxy substituents, polarized electrostatic distribution, and modifiable C6 position constitute the major pharmacophoric and structurally optimizable features of DHSA, providing a theoretical basis for the rational design and future optimization of DHSA derivatives.

## 5. Toxicity of DHSA

Toxicological evaluation is critical for the clinical development of DHSA. Although its biosynthetic precursor, SA, exhibits potent anticancer, anti-inflammatory, and antimicrobial activities, increasing evidence indicates that SA induces multi-organ toxicity involving the cardiovascular, nervous, immune, hepatic, and developmental systems, thereby substantially limiting its clinical application [85,86,87,88]. Recent toxicological studies showed that acute oral administration of SA resulted in LD_50_ values of 1000 mg/kg in male Sprague–Dawley rats and 926 mg/kg in female rats, classifying SA as a GHS Category 4 toxic compound [89]. Subacute exposure further induced multi-organ injury, including pulmonary hemorrhage, hepatic steatosis, renal tubular necrosis, myocardial hemorrhage, interstitial pneumonia, and intestinal epithelial damage, accompanied by redox imbalance and gut microbiota dysbiosis [89]. Female rats also showed greater sensitivity to SA-induced toxicity [89]. Toxicokinetic analysis further revealed hepatic and renal tissue accumulation, nonlinear elimination kinetics, and prolonged systemic exposure, which may contribute to its cumulative toxicity [89].

In zebrafish and HL1 cardiomyocytes, SA exposure induced severe cardiotoxicity characterized by cardiac malformations, reduced heart rate, and impaired blood flow [90]. Developmental toxicity studies further showed that SA caused embryonic malformations, shortened body length, enlarged yolk sacs, and impaired vascular development in zebrafish larvae [91]. In addition to zebrafish developmental toxicity, the embryotoxic effects of SA in mammalian systems have also attracted attention. In mouse blastocysts, SA exposure significantly increased apoptosis and reduced inner cell mass (ICM) cell numbers, leading to impaired blastocyst viability and inhibited embryonic cell proliferation [92]. SA-treated blastocysts further showed reduced implantation capacity, increased embryo resorption, decreased fetal survival, and lower fetal weight in vivo, indicating severe impairment of post-implantation embryonic development [92]. In addition to cardiotoxicity and developmental toxicity, SA also exhibits significant immunotoxicity and neurotoxicity [93,94]. SA exposure reduced neutrophil and macrophage numbers in zebrafish larvae, impaired innate immune cell migration, and disrupted locomotor behavior [93]. Neurotoxicity studies further confirmed that SA induces neuronal apoptosis and cerebrovascular injury, highlighting its broad systemic toxicity [94].

Compared with SA, DHSA exhibits a markedly improved safety profile while retaining considerable antitumor activity. DHSA showed cytotoxic activity against BEL-7402 hepatoma cells and HeLa cervical carcinoma cells, with IC_50_ values of 36.77 ± 2.19 and 27.14 ± 0.96 μg/mL, respectively, while showing no significant cytotoxicity toward normal human skin epidermal cells (IC_50_ > 50 μg/mL) [95]. In HL-60 promyelocytic leukemia cells, DHSA displayed substantially lower cytotoxicity than SA. After 24 h exposure to 20 μM DHSA, cell viability remained approximately 52%, whereas SA induced marked cytotoxicity at submicromolar concentrations [96]. Importantly, subchronic toxicity studies further support the favorable in vivo safety profile of DHSA. Continuous dietary administration of DHSA to Wistar rats for 90 days at doses up to 58 mg/kg/day caused no significant changes in body weight, organ morphology, histopathology, hematological parameters, serum biochemical indices, oxidative stress markers, or DNA damage [97]. After prolonged exposure, no apparent hepatic DNA adduct formation or abnormal modulation of cytochrome P450 activity was detected [97]. Collectively, these findings suggest that DHSA retains considerable antitumor efficacy while exhibiting substantially lower systemic toxicity and a potentially wider therapeutic window than SA.

**Figure 5:**
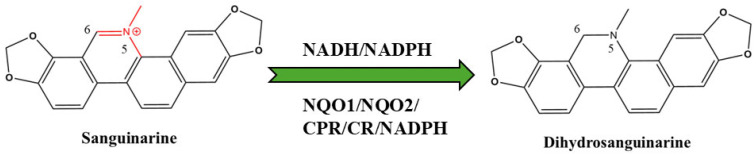
Metabolic conversion of sanguinarine (SA) to dihydrosanguinarine (DHSA). SA undergoes enzymatic and non-enzymatic reduction of the C5–C6 imine bond to form DHSA. Hepatocellular cofactors NADH and NADPH, together with reductases including NADPH quinone oxidoreductase 1 (NQO1), NQO2, cytochrome P450 reductase (CPR), and carbonyl reductase (CR), participate in this reduction process. The conversion eliminates the quaternary ammonium cation and reduces molecular planarity, which may contribute to the lower toxicity profile of DHSA. The red part indicates the reduction site of SA.

The reduced toxicity of DHSA is closely associated with its structural and metabolic characteristics. Structurally, SA contains a rigid planar benzophenanthridine scaffold with a permanently charged quaternary ammonium cation, which promotes strong interactions with DNA and facilitates DNA intercalation, oxidative damage, and genotoxicity [98]. In contrast, DHSA is generated through two-electron reduction of the C5–C6 imine bond of SA into a single bond, thereby eliminating the quaternary ammonium cation and reducing molecular planarity [99,100]. This structural conversion weakens DNA intercalation and nonspecific nucleic acid interactions, which may contribute to the lower toxicity of DHSA compared with SA [101,102,103,104]. The structural comparison between SA and DHSA is shown in Figure 5. Interestingly, SA can be metabolically converted into DHSA through both enzymatic and non-enzymatic reduction pathways. Low-molecular-weight reductants, particularly the hepatocellular cofactors NADH and NADPH, can reduce the C5–C6 imine bond of SA into a single bond [99,103,104]. In parallel, several hepatic enzymes, including NADPH quinone oxidoreductase 1 (NQO1), NQO2, cytochrome P450 reductase (CPR), and carbonyl reductase (CR), participate in this conversion process [98,105] (Figure 5). The resulting DHSA retains the key phenanthridine pharmacophore of SA while undergoing charge neutralization, thereby improving lipophilicity and membrane permeability. This structural alteration may improve tissue distribution properties while reducing nonspecific hepatorenal toxicity in vivo [100]. Therefore, metabolic conversion of SA into DHSA may represent an intrinsic detoxification process that generates a less toxic metabolite while preserving the essential pharmacophore of SA.

Current toxicological evidence of DHSA still mainly focuses on preliminary cytotoxicity and short-term safety assessments, whereas systematic comparative evaluations of key toxicological parameters, including LD_50_, therapeutic index, and dose–response relationships for hepatotoxicity, cardiotoxicity, neurotoxicity, and long-term systemic toxicity, are still lacking. Therefore, although existing indirect evidence suggests that DHSA exhibits lower toxicity than SA, comprehensive and standardized in vivo comparative toxicological studies are still required to definitively establish the safety advantages and clinical translational potential of DHSA-based therapeutics.

## 6. Pharmacokinetics of DHSA

### 6.1. In Silico ADME Prediction

Predictive analysis using SwissADME (http://www.swissadme.ch/) (accessed on 1 March 2026) indicated that DHSA complies with Lipinski’s rule of five, including a molecular weight ≤ 500, hydrogen-bond donors ≤ 5, hydrogen-bond acceptors ≤ 10, and a logP value ≤ 5. These physicochemical characteristics suggest a favorable balance between lipophilicity and aqueous solubility, which is considered a prerequisite for passive diffusion across intestinal epithelial cells, thereby implying potentially high gastrointestinal absorption of DHSA. In addition, the rigid planar aromatic scaffold and moderate lipophilicity of DHSA may facilitate passive diffusion across biological membranes, including the blood–brain barrier. Furthermore, DHSA was predicted to inhibit several cytochrome P450 enzymes, including CYP1A2, CYP2C19, CYP2C9, and CYP3A4. The predicted skin permeation coefficient (LogKp) was −5.41 cm/s, further supporting its membrane permeability profile. Overall, the preliminary pharmacokinetic properties of DHSA suggest acceptable oral bioavailability and favorable drug-likeness. Given that DHSA inhibits multiple CYP isoforms, co-administration with substrates of these enzymes, such as warfarin (CYP2C9), omeprazole (CYP2C19), or various chemotherapeutic agents (CYP3A4), may alter drug clearance and increase the risk of adverse effects [106,107,108,109]. Conversely, CYP inducers or inhibitors may also affect the pharmacokinetics of DHSA itself. To date, no in vivo drug–drug interaction studies involving DHSA have been reported. Therefore, future investigations should evaluate whether DHSA may act as either a perpetrator or a victim of CYP-mediated interactions, particularly if it is to be developed as an adjunctive therapeutic agent [109].

### 6.2. Pharmacokinetic Studies in Animal Models

Currently available pharmacokinetic studies of DHSA in animal models remain relatively limited and are characterized by substantial heterogeneity in animal species, administration routes, dosages, and experimental designs (Table 3). Importantly, most reported DHSA pharmacokinetic parameters were obtained from metabolite exposure after SA administration rather than following direct DHSA dosing. Therefore, caution is required when directly comparing the pharmacokinetic profiles of DHSA and SA, because metabolite exposure does not necessarily reflect the intrinsic disposition characteristics of DHSA itself.

To date, direct pharmacokinetic evaluation following DHSA administration has only been reported in male Wistar rats after oral administration of DHSA at 91 mg/kg. In this study, DHSA reached peak plasma concentration at approximately 1 h after administration, and multiple plasma concentration peaks were observed during the absorption phase, suggesting the presence of enterohepatic circulation [97]. Interestingly, this phenomenon disappeared following bile duct ligation, indicating that enterohepatic circulation may contribute to prolonged systemic exposure of DHSA [97]. In addition, DHSA was predominantly detected in fecal samples rather than urine, further supporting the involvement of biliary excretion and intestinal metabolism in its disposition [97]. Compared with rapidly eliminated alkaloids, these characteristics may favor sustained systemic retention and prolonged pharmacological activity. Recent studies further suggest that prolonged intestinal exposure of DHSA may also contribute to the modulation of gut microbiota composition and intestinal immune homeostasis [110]. In broiler models, DHSA increased the abundance of Lactobacillus species, promoted the production of indole-derived tryptophan metabolites and short-chain fatty acids, activated the aryl hydrocarbon receptor (AhR) pathway, enhanced intestinal barrier integrity, and attenuated inflammatory responses [110]. Collectively, these findings suggest that the enterohepatic circulation and intestinal retention characteristics of DHSA are closely associated with its gut microbiota–immune regulatory effects and may contribute to its anti-inflammatory and metabolic activities.

Current evidence indicates that DHSA is a primary metabolite formed during the biotransformation of SA in vivo [18]. The in vivo pharmacokinetic behavior of DHSA and SA is markedly influenced by the route of administration. Following intramuscular administration of SA in pigs (0.1 mg/kg), SA and DHSA exhibited identical T_max_ values (0.25 h), while C_max_ values were 30.16 ng/mL and 5.61 ng/mL (approximately 5.4-fold difference), and AUC_0–24_ values were 35.40 ng·h/mL and 10.18 ng·h/mL, respectively. The corresponding T_1/2_ values were 1.29 h for SA and 1.90 h for DHSA, indicating that SA drives rapid and high systemic exposure, whereas DHSA, as an early-phase metabolite, displays a longer elimination phase, suggesting a more sustained exposure profile that may be advantageous for chronic disease management. Following oral administration of SA in pigs (0.1 mg/kg), T_max_ was prolonged to 2.75 h, while C_max_ decreased to 3.40 ng/mL (approximately 89% reduction) and AUC to 15.62 ng·h/mL (approximately 56% reduction); for DHSA, C_max_ was 2.41 ng/mL (approximately 57% reduction) and AUC was 9.12 ng·h/mL (approximately 10% reduction), with T_1/2_ values of 2.33 h and 2.20 h for SA and DHSA, respectively. These results suggest that oral administration markedly reduces SA exposure due to a pronounced first-pass effect while enhancing SA-to-DHSA metabolic conversion, resulting in relatively more stable DHSA exposure and indicating a potential pharmacokinetic advantage of DHSA under clinically relevant oral conditions. Under repeated oral dosing (0.1 mg/kg, three times daily for 3 consecutive days), SA exposure increased substantially, with C_max_ rising from 3.40 to 5.86 ng/mL (≈72%) and AUC from 15.62 to 31.06 ng·h/mL (≈99%), accompanied by an increase in T_1/2_ from 2.33 to 3.17 h; in contrast, DHSA showed more moderate increases in C_max_ (2.41 to 2.95 ng/mL, ≈22%) and AUC (9.12 to 13.47 ng·h/mL, ≈48%), while T_1/2_ remained relatively stable (2.20 to 2.38 h). The accumulation index was 1.21 for SA and 1.11 for DHSA, indicating limited accumulation overall, with DHSA exhibiting a lower propensity for accumulation and more stable elimination kinetics, suggesting a reduced risk of accumulation-related toxicity during long-term administration [19]. It is worth noting that significant species differences in DHSA pharmacokinetics were observed: in broiler chickens following oral administration of SA, DHSA reached a C_max_ of 5.17 ng/mL and AUC of 21.79 ng·h/mL, exceeding SA-related exposure patterns (0.90 ng/mL and 45.19 ng·h/mL), indicating substantially higher SA-to-DHSA conversion efficiency in chickens than in pigs. Collectively, these data demonstrate that non-oral administration is characterized by SA-dominated rapid exposure with DHSA acting as a secondary metabolite with prolonged persistence, whereas oral administration enhances metabolic conversion to DHSA and yields relatively stable exposure profiles; repeated dosing further increases exposure modestly without significant accumulation. Overall, the pharmacokinetic characteristics of DHSA support its potential as a promising orally administered candidate for chronic disease therapy, although further systematic studies are required to validate its translational advantage over SA.

**Table 3:** Pharmacokinetic parameters of sanguinarine (SA) and dihydrosanguinarine (DHSA) reported in different animal models.

Animal Model	Administration Method and Dosage	PK Source Type	Analyte	T_1/2_ (h)	T_max_ (h)	C_max_ (ng/mL)	AUC_0–24_ (ng·h/mL)	AUC_0–∞_ (μg·h/L)	Reference
Male Wistar rats	Oral administration of DHSA (91 mg/kg)	Direct DHSA administration	DHSA	NR	1.0	28.08	NR	NR	[97]
Broiler chickens	Oral administration of SA (30 mg/kg)	DHSA detected as metabolite after SA administration	SA	4.20 ± 0.43	0.38 ± 0.30 h	0.90 ± 1.05	45.19 ± 3.54	NR	[111]
			DHSA	4.29 ± 0.64	0.25 ± 0.12	5.17 ± 0.34	21.79 ± 7.36	NR	
Six-week-old yellow-feathered broiler chickens	Oral administration of Sangrovit^®^ (20 mg/kg, equivalent to SA 0.384 mg/kg)	DHSA detected as metabolite after SA administration	SA	1.05 ± 0.19	0.90 ± 0.65	1.89 ± 0.88	NR	9.92 ± 5.46	[112]
			DHSA	0.83 ± 0.11	0.59 ± 0.39	2.49 ± 1.41	NR	6.08 ± 3.49	
Broiler chickens	Intravenous administration of SA (0.038 mg/kg)	DHSA detected as metabolite after SA administration	SA	0.34 ± 0.13	NR	NR	NR	4.45 ± 0.40	[112]
			DHSA	1.12 ± 0.29	NR	NR	NR	7.81 ± 1.12	
Female pigs (7–8 weeks old)	Intramuscular administration of SA (0.1 mg/kg)	DHSA detected as metabolite after SA administration	SA	1.29 ± 0.21	0.25 ± 0.00	30.16 ± 5.85	35.40 ± 4.26	NR	[19]
			DHSA	1.90 ± 0.19	0.25 ± 0.00	5.61 ± 0.73	10.18 ± 1.81	NR	
Female pigs (7–8 weeks old)	Single oral administration of SA (0.1 mg/kg)	DHSA detected as metabolite after SA administration	SA	2.33 ± 0.11	2.75 ± 0.27	3.40 ± 0.36	15.62 ± 1.29	NR	[19]
			DHSA	2.20 ± 0.12	2.75 ± 0.27	2.41 ± 0.24	9.12 ± 0.59	NR	
Female pigs (7–8 weeks old)	Multiple oral administration of SA (0.1 mg/kg, three times daily for 3 days)	DHSA detected as metabolite after SA administration	SA	3.17 ± 0.22	2.58 ± 0.20	5.86 ± 0.98	31.06 ± 3.38	NR	[19]
			DHSA	2.38 ± 0.17	2.67 ± 0.26	2.95 ± 0.36	13.47 ± 1.79	NR	

NR, not reported. DHSA parameters in several studies were obtained as metabolite exposure after SA administration rather than following direct DHSA dosing. Original pharmacokinetic units reported in the cited studies were retained.

## 7. Clinical Application Prospects and Challenges

Although DHSA exhibits broad pharmacological activities and favorable drug-development potential, its clinical translation still faces multiple substantial challenges. As a planar benzo[c]phenanthridine alkaloid with pronounced multi-target properties, DHSA represents a typical “double-edged sword” in natural product drug discovery. While its pleiotropic regulatory effects provide therapeutic advantages in complex diseases characterized by dysregulated inflammatory and oncogenic signaling networks, these same characteristics also increase the risk of off-target effects, systemic toxicity, and unpredictable pharmacodynamic responses across different pathological contexts [10,20]. Therefore, future translational studies should place greater emphasis on systematically defining the therapeutic window and tissue-specific safety profile of DHSA.

Particular caution is warranted regarding the bidirectional regulatory effects of DHSA on p53 signaling. Current evidence indicates that DHSA exerts distinct regulatory effects on mutant and wild-type p53 proteins depending on the characteristics of different tumor cells [15]. Therefore, identification of appropriate tumor patient populations through biomarker-guided screening strategies represents a feasible approach for the future clinical application of DHSA [113]. First, TP53 sequencing should be performed to determine whether tumors harbor wild-type TP53, missense mutations, truncating mutations, or TP53 deletion [114]. Second, p53 protein expression in tumor tissues should be evaluated by immunohistochemistry, because strong nuclear accumulation of p53 often indicates stabilization of mutant p53, whereas complete loss of p53 staining may suggest nonsense mutations, frameshift mutations, or TP53 deletion [115]. Third, the functional status of the p53 pathway should be assessed by examining downstream p53-responsive markers, such as p21, BCL-2-associated X protein (BAX), p53 upregulated modulator of apoptosis (PUMA), and murine double minute 2 (MDM2), in tumor tissues or biopsy specimens, in order to distinguish tumors retaining functional p53 signaling from those with functionally inactive p53 pathways [116,117]. Based on these biomarker profiles, assessment of TP53 mutation status, p53 protein expression, and p53 pathway activity provides a practical basis for selecting patient populations suitable for DHSA-based therapies.

In addition, the multitarget nature of DHSA may increase the risk of systemic toxicities involving multiple organs and signaling networks, thereby necessitating comprehensive evaluation and management of potential adverse effects and drug–drug interaction risks [118]. Future investigations should therefore combine multi-organ toxicity evaluation, cytochrome P450 interaction profiling, transporter-mediated pharmacokinetic analyses, and systems pharmacology approaches in order to characterize the tissue-specific safety boundaries and potential interaction networks of DHSA in vivo. Particular attention should be paid to evaluating dose-dependent toxicities and differences in organ susceptibility to DHSA-related toxic effects during chronic administration or combination therapy [119]. In addition, optimization of dosage regimens and biomarker-guided safety monitoring may help reduce systemic exposure-related adverse effects while improving the therapeutic selectivity of DHSA.

From an active pharmaceutical ingredient (API) development perspective, establishment of a robust, economical, and sustainable supply chain remains a non-negligible challenge for the clinical translation of DHSA [120]. Because DHSA is primarily derived from natural plant resources, variations in plant origin, cultivation conditions, harvesting seasons, and extraction efficiency may significantly affect product yield, purity, and batch-to-batch consistency. Therefore, future studies should focus on establishing standardized upstream production systems for DHSA, including the optimization of extraction and purification procedures as well as the establishment of quality control standards for raw materials and intermediates. Meanwhile, development of scalable semi-synthetic or total synthetic strategies will help reduce the dependence of DHSA on plant-derived resources while improving production stability. Furthermore, the implementation of quality-by-design (QbD)-based manufacturing frameworks integrating critical process parameters, impurity profiling, process analytical technologies, and batch consistency monitoring will be essential for ensuring the reproducibility, stability, and pharmaceutical quality of DHSA products during large-scale production [121].

## 8. Conclusions and Perspective

Dihydrosanguinarine (DHSA), a reduced benzophenanthridine alkaloid derived from sanguinarine, represents a promising natural small-molecule scaffold with broad pharmacological activities and relatively favorable safety characteristics. Current evidence demonstrates that DHSA exerts antitumor, anti-inflammatory, hypoglycemic, neuroprotective, analgesic, anxiolytic, antiarrhythmic, and antimicrobial effects through modulation of multiple signaling pathways and molecular targets, including TMEM16A, p53, Ras/Raf/MEK/ERK, PI3K/AKT, NF-κB, PPARγ, GABA_A_ receptors, and voltage-gated sodium channels [7,10,15]. Existing evidence further predicts that DHSA functions as a multitarget alkaloid rather than a highly selective single-target agent. This pharmacological characteristic may provide unique therapeutic advantages for complex diseases involving intertwined inflammatory, metabolic, oncogenic, and neurophysiological pathways, while also increasing the risk of off-target toxicity of DHSA [122]. Current pharmacokinetic studies indicate that DHSA possesses acceptable membrane permeability, gastrointestinal absorption potential, enterohepatic circulation characteristics, and relatively sustained systemic exposure, making it highly worthy of development as an oral drug for the treatment of chronic diseases. Compared with its biosynthetic precursor SA, DHSA exhibits lower systemic toxicity while retaining considerable biological activity, thereby highlighting its further developmental potential [97]. In addition, SAR and ESP analyses suggest that the rigid aromatic scaffold and methylenedioxy substituents together constitute the structural basis underlying the broad-spectrum biological activities of DHSA, whereas chemical modification at the C6 position provides a feasible direction for subsequent drug optimization and structural refinement.

Nevertheless, despite these promising findings, the clinical translation of DHSA still faces several critical challenges. Current studies remain fragmented and are largely limited to preliminary in vitro observations, whereas systematic in vivo target validation, tissue-specific analyses, and biomarker-guided therapeutic stratification remain insufficient. Meanwhile, the multitarget nature of DHSA may also increase the risk of off-target toxicities and context-dependent pharmacological responses [123]. Before successful clinical application can be achieved, several additional bottlenecks must also be addressed, including limited aqueous solubility despite acceptable membrane permeability, incomplete toxicological evaluation, and the lack of standardized large-scale manufacturing and quality control systems.

Therefore, optimization strategies targeting the pharmacokinetic and safety limitations of DHSA remain particularly important for its future translational development. Novel drug delivery systems (NDDS) have demonstrated considerable potential for improving the solubility, bioavailability, stability, and tissue-targeting efficiency of natural compounds [124,125]. Nanotechnology-based formulations, including nanosuspensions, nanoliposomes, micelles, self-microemulsifying systems, nanocapsules, and solid lipid nanoparticles, may all represent promising strategies for optimizing DHSA delivery [126,127]. In addition, rational formulation design may help reduce systemic exposure while improving therapeutic selectivity, thereby partially mitigating DHSA-associated systemic toxicity risks. For example, pulmonary-targeted nanoformulations may represent an attractive strategy for the application of DHSA in respiratory inflammatory diseases such as acute lung injury [128]. Meanwhile, GLP-compliant long-term toxicological studies, standardized pharmacokinetic evaluations, and QbD-oriented manufacturing strategies will also be essential for the future clinical translation of DHSA [129].

Overall, DHSA should not merely be regarded as a reduced metabolite of SA, but rather as a biologically active alkaloid possessing unique pharmacological, toxicological, and pharmacokinetic characteristics. Although substantial challenges still remain before successful clinical translation can be achieved, continued multidisciplinary efforts integrating medicinal chemistry, systems pharmacology, toxicology, pharmaceutical engineering, and advanced drug delivery technologies are expected to further promote the development of DHSA from a bioactive natural alkaloid into a clinically translatable lead compound or therapeutic candidate for complex human diseases.

## Figures and Tables

**Table 1:** Physicochemical properties of DHSA.

Name	Dihydrosanguinarine
Chemical Structure	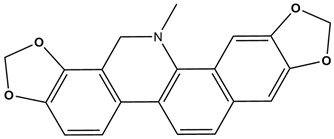
CAS	3606-45-9
Molecular formula	C_20_H_15_NO_4_
Molecular weight	333.3 g/mol
Color	White to pale yellow
Melting point	190–191 °C
Boiling point	566.9 ± 29.0 °C
Solubility	DMSO: 5.2 mg/mL (needs ultrasound and heating)
Density	1.426 ± 0.06 g/cm^3^
Acidity coefficient (pKa)	2.36 ± 0.20

**Table 2:** The pharmacological activities and mechanisms of dihydrosanguinarine (DHSA).

Pharmacological Activities	Experimental Model	Study Type	Dose/Concentration	Biological Effects	Mechanisms	Reference
Anti-lung adenocarcinoma	LA795 cells	In vitro	3–10 μM	↓TMEM16A	Induction of apoptosis via suppression of TMEM16A chloride currents	[20]
Anti-pancreatic cancer	PANC-1 cells	In vitro	10–20 μM	↓mut-Ras, ↓mut-p53, ↓p-c-Raf, ↓p-ERK	Induction of apoptosis and cell cycle arrest via inhibition of Ras/Raf/MEK/ERK signaling and bidirectional regulation of p53	[15]
	SW1990 cells	In vitro	10–20 μM	↑WT-Ras, ↑WT-p53, ↓p-c-Raf, ↓p-ERK		
Anti-inflammatory effect	LPS induced male Balb/C mice	In vivo	10–20 mg/kg	↓F4/80, ↓TNF-α, ↓IL-1β, ↓IL-6, ↓CXCL1/2/3, ↓IL-17A, ↓IL-17RA, ↓IL-17RE, ↓TRAF3IP2, ↓p-AKT	Suppression of macrophage infiltration and TNF/IL-17 signaling	[10]
	LPS induced RAW 264.7	In vitro	10–100 μg/mL	↓iNOS, ↓TNF-α, ↓IL-1β, ↓IL-6, ↓p-IκBα, ↓p-p65	Inhibition of NF-κB signaling	[21]
	LPS induced male Balb/C mice	In vivo	1–4 g/kg	↓NO, ↓IL-1β, ↓iNOS	Reduction in inflammatory mediator production	[21]
Anti-type 2 diabetes effect	3T3-L1 cells	In vitro	5 μM	↓AMPK, ↓RXR, ↓p-PPARγ, ↑aP2, ↑CD36, ↑C/EBPα, ↑Glut4, ↑adiponectin	Modulation of the AMPKα/PPARγ/GLUT4 pathway	[16]
Analgesic Effect	Male Swiss Webster mice	In vivo	100 mg/kg	/	Modulating the GABA_A_ and opioid receptors.	[12]
	CHO cells expressing human Nav1.7	In vitro	20 μM	Inhibition of Nav1.7 sodium currents	Binding to the Nav1.7 pore region and inhibition of sodium influx	[22]
Anti-arrhythmic Effect	CHO cells expressing human Nav1.5	In vitro	32 μM	Inhibition of Nav1.5 sodium currents	Binding to the Nav1.5 pore domain and inhibition of sodium influx	[22]
Anxiolytic effect	Male Balb/c mice	In vivo	0.1–10 mg/kg	Activation of GABA_A_ receptors	GABA_A_ receptor activation	[7]

↓, down regulated; ↑, up regulated.

## Data Availability

No new data were created or analyzed in this study.

## Abbreviations

8-OHdG	8-Hydroxy-2′-deoxyguanosine
AIC	Arrhythmia-induced cardiomyopathy
AKT	Protein kinase B
AMPK	AMP-activated protein kinase
aP2	Adipocyte protein 2
API	Active pharmaceutical ingredient
BAX	BCL-2-associated X protein
C/EBPα	CCAAT enhancer binding protein alpha
CAT	Catalase
CD36	Cluster of differentiation 36
Cdc25c	Cell division cycle 25C
CDK	Cyclin-dependent kinase
CPR	Cytochrome P450 reductase
CR	Carbonyl reductase
CXCL	C-X-C motif chemokine ligand
DHSA	Dihydrosanguinarine
ERK	Extracellular regulated protein kinases
ESP	Electrostatic surface potential
GABA_A_	γ-Aminobutyric acid type A
GPx	Glutathione Peroxidase
GSH	Glutathione
ICM	Inner cell mass
IκBα	Nuclear factor kappa-B inhibitor alpha
JNK	c-Jun N-terminal kinase
LUAD	Lung adenocarcinoma
LVSD	Left ventricular systolic dysfunction
MAPK	Mitogen-activated protein kinase
MDA	Malondialdehyde
MDM2	Murine double minute 2
MEK	Mitogen-activated protein kinase kinase
mut-p53	Mutant p53
NDDS	Novel drug delivery systems
NF-κB	Nuclear factor kappa-B
NQO1	NADPH quinone oxidoreductase 1
Nrf2	Nuclear factor erythroid 2–related factor 2
NTX	Naltrexone
p38	p38 Mitogen-activated protein kinase
PI3K	Phosphatidylinositol 3-kinase
PPARγ	Peroxisome proliferator-activated receptor gamma
PTX	Picrotoxin
PUMA	p53 upregulated modulator of apoptosis
QbD	Quality-by-design
Raf	Raf kinases
Ras	Rat sarcoma
ROS	Reactive oxygen species
RXR	Retinoid X receptor
SA	Sanguinarine
SAR	Structure–activity relationship
SOD	Superoxide dismutase
TNF-α	Tumor necrosis factor-α
VGSCs	Voltage-gated sodium channels
WT-p53	Wild-type p53
